# The Effect of Intravenous Iron Treatment on Quality of Life in Inflammatory Bowel Disease Patients with Nonanemic Iron Deficiency

**DOI:** 10.1155/2015/582163

**Published:** 2015-01-26

**Authors:** Cem Çekiç, Serkan İpek, Fatih Aslan, Zehra Akpınar, Mahmut Arabul, Firdevs Topal, Elif Sarıtaş Yüksel, Emrah Alper, Belkıs Ünsal

**Affiliations:** Department of Gastroenterology, İzmir Atatürk Training and Research Hospital, Izmir Kâtip Çelebi University, Karabaglar, 35160 İzmir, Turkey

## Abstract

*Background.* Iron deficiency is the prevalent complication of inflammatory bowel disease (IBD). Herein, we investigated the effect of intravenous iron treatment on quality of life (QoL) in nonanemic and iron deficient IBD patients.* Methods.* Eighty-five IBD patients were recruited for this study. The patients were intravenously administered 500 mg iron sucrose in the first week of the study. Hematologic parameters and QoL were evaluated before to iron treatment and during the 12th week of treatment. The Inflammatory Bowel Disease Questionnaire (IBDQ) and the Short Form-36 (SF-36) Health Survey were used to assess QoL.* Results.* Prior to intravenous iron administration, the IBDQ, SF-36 Physical Component Summary (PCS) and Mental Component Summary (MCS) scores were 152.3 ± 30.6, 46.7 ± 7.3, and 45.7 ± 9.8, respectively. In the 12th week of iron administration, those scores were 162.3 ± 25.5 (*P* < 0.001), 49.3 ± 6.4 (*P* < 0.001), and 47.6 ± 8.9 (*P* = 0.024), respectively, which were all significantly different from the scores prior to iron administration. The mean changes in the IBDQ scores for ulcerative colitis and Crohn's disease were 8.7% and 3.0% (*P* = 0.029), were 6.4% and 4.7% (*P* = 0.562) for the SF-36 PCS, and were 4.6% and 3.2% (*P* = 0.482) for the SF-36 MCS, respectively.* Conclusion.* Intravenous iron treatment may improve QoL in nonanemic, but iron deficient, IBD patients.

## 1. Introduction

Anemia and iron deficiency (ID) are some of the most prevalent extraintestinal complications of IBD. Approximately one-third of IBD patients are anemic, and 45% of IBD patients are iron deficient. Coexisting subclinical inflammation and minor histologic abnormalities usually play a role in iron deficiency (probably through the hepcidin-ferroportin axis) even though the patients are in remission. Additionally, IBD patients are not treated properly for iron deficiency during disease activation and the iron deficiency status usually does not recover even if remission is achieved [1]. Iron is an essential element for the proper function of hemoglobin, myoglobin, cytochromes, and several enzymes. It plays a crucial role in various metabolic processes, including oxidative phosphorylation, mitochondrial functions, neurotransmitter synthesis, and erythropoiesis. Because of iron's importance, ID causes malaise, reduced exercise capacity, cognitive dysfunction, and hematologic complications [2]. In addition, anemia and ID have been shown to disrupt QoL in IBD patients [3].

According to current literature, intravenous iron replacement is the best way to treat ID in IBD patients [4]. Intravenous iron replacement is more efficient and tolerable than oral administration, and it has been shown to be more effective in improving QoL [5]. In recent years, treatment of iron deficiency anemia in IBD patients has been defined, but there is still no consensus on treating those with nonanemic iron deficiency (NAID) [6]. NAID has been shown to decrease QoL in patients with congestive heart failure, cyanotic cardiac diseases, and restless leg syndrome, while intravenous iron replacement has been shown to improve QoL in those with these conditions [7, 8].

There is limited research investigating the effect of iron replacement therapy on QoL in IBD patients. Herein, we investigated the efficacy of intravenous iron treatment on hematological parameters and QoL in IBD patients with NAID. WHO criteria for anemia are as follows: hemoglobin level is lower than 13 g/dL (130 g/L) in men and 12 g/dL (120 g/L) in women.

## 2. Patients and Methods

### 2.1. Patients

Eighty-five IBD patients with ID who were in remission (proven by clinical and biochemical parameters) were included in this study.

#### 2.1.1. Inclusion Criteria

Inclusion criteria are as follows: C-reactive protein <5 mg/L, Crohn's disease activity index <150 for those with Crohn's disease (CD), and modified Truelove and Witts' index ≤3 for those with ulcerative colitis (UC) [9, 10]. Females with Hb >12 g/dL, males with Hb >13 g/dL, and those with ferritin <30 mcg/L or transferrin saturation (TfS) <16% were diagnosed as NAID [11]. The study included also those older than 18 years and those in the reproductive period with a negative urine pregnancy test who agreed to use a reasonable contraception method during the study period.

#### 2.1.2. Exclusion Criteria

Low vitamin B_12_ and folic acid levels, severe cardiopulmonary, hepatic or renal diseases, history of intravenous or oral iron administration or blood transfusion in the last month, history of iron allergy, diagnosis of psychiatric disease or antipsychotic drug usage, malignancy and autoimmune diseases, pregnancy or lactation, medical modification applied for IBD in the last month or during the study period, and IBD exacerbation during the study period are criteria for exclusion.

The Montreal classification was used to indicate the disease localization and behavior in both UC and CD patients [12].

### 2.2. Study Design

Five hundred mg iron sucrose (FeS) (Venofer) was administered per week in three fragments (200-200-100 mg) to IBD patients that were in remission and who fulfilled the NAID criteria. This fixed amount was decided according to the Ganzoni formula since it recommends administering 500 mg to replace iron storage. FeS was infused in 100 mL isotonic sodium chloride for 30 minutes. No test dose or premedication was administered prior to the infusion. Hematologic parameters were recorded, and QoL questionnaires were evaluated prior to treatment and during the 12th week of treatment.

### 2.3. Hematologic Assessment

Hemoglobin, serum iron, ferritin, and TfS were determined prior to treatment and during the 12th week of the intravenous iron administration. We evaluated the response to treatment during the 12th week based on previous studies performed with FeS [13].

### 2.4. QoL Assessment

The Inflammatory Bowel Disease Questionnaire (IBDQ) and the SF-36 Health Survey (version 2) were used to evaluate the difference in QoL before intravenous iron administration and during the 12th week. IBDQ is a specific index for both UC and CD that is used in the majority of UC and CD related research [14]. It has 32 questions, each of which uses a Likert scale [1–7]. The total score varies between 32 and 224, with the highest score indicating the best QoL. In our study, norm-based scores for the SF-36 survey were taken into consideration. In norm-based scoring, the general population has a mean score of 50 and a standard deviation of 10 [15]. We evaluated the Physical Component Summary (PCS) and the Mental Component Summary (MCS) for the SF-36 survey. Turkish versions of both questionnaires are validated, and a disbursement for the copyright was made with the order number 12751 to the copyright holder, McMaster University.

### 2.5. Statistical Analysis

All statistical analyses were performed using SPSS for Windows 15 (SPSS Inc., Chicago). Descriptive statistics were represented as *n* (%), median (IQR), and mean ± SD. Baseline and follow-up hematologic parameters and QoL scores were compared using the paired samples *t*-test and the Wilcoxon signed rank test. The Mann-Whitney *U* Test was used to determine changes in QoL scores among different disease types. A 2-sided *P* value of 0.05 was considered statistically significant.

### 2.6. Ethical Considerations

The Ethics Committee of Katip Çelebi University Faculty of Medicine, Izmir, Turkey approved this study (date: September 27, 2013; Project number 197). The patients were included into the study after obtaining informed written consent.

## 3. Results

### 3.1. Patient Characteristics

Eighty-five IBD patients (55 with UC and 30 with CD) were prospectively included in this study. Their mean age was 42.2 ± 13.1 years, and 54.1% (*n* = 46) were males. Their median IBD duration time was 4 years. Seventy-five patients out of 85 (91.7%) had ferritin <30 mcg/L and 53 (62.3%) patients had TfS <16%. None of the patients dropped out from the study. UC and CD localizations, CD behavior, and these demographic data are presented in Table 1.

### 3.2. Changes in the Hematologic Parameters

**Figure 1:**
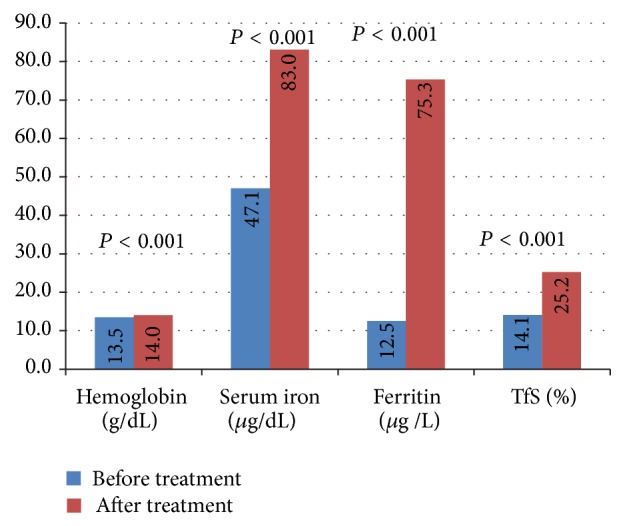
Mean hematologic parameters before and after intravenous FeS treatment.

The hematologic parameters before intravenous iron administration and during the 12th week of administration were as follows, respectively: hemoglobin (g/dL), 13.5 ± 1.0, 14.0 ± 1.0 (*P* < 0.001); serum iron (*μ*g/dL), 47.1 ± 26.6, 83.0 ± 32.6 (*P* < 0.001); ferritin (*μ*g/L), 12.5 ± 6.7, 75.3 ± 43.9 (*P* < 0.001); and TfS (%), 14.1 ± 9.1, 25.2 ± 10.5 (*P* < 0.001). These data are presented in Figure 1.

### 3.3. Changes in the QoL Measures

The QoL measures before intravenous iron administration and during the 12th week of administration were, respectively, as follows: IBDQ scores, 152.3 ± 30.6 and 162.3 ± 25.5 (*P* < 0.001); SF-36 PCS, 46.7 ± 7.3 and 49.3 ± 6.4 (*P* < 0.001); and SF-36 MCS, 45.7 ± 9.8 and 47.6 ± 8.9 (*P* = 0.024).

**Figure 2:**
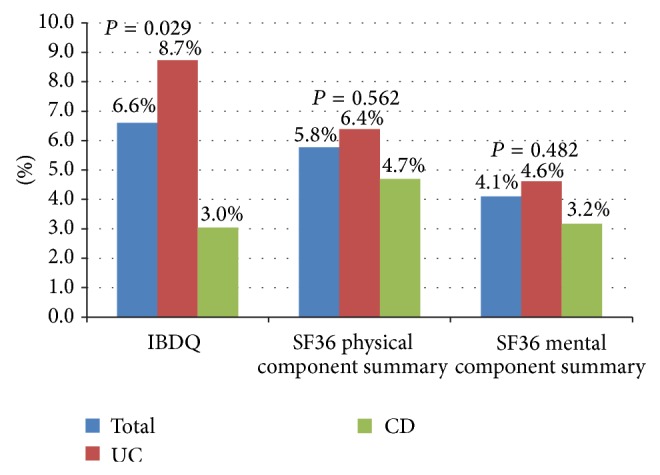
Percentage changes according to baseline in QoL scores.

The QoL measures before and during the 12th week of intravenous iron administration for UC patients are as follows, respectively: IBDQ, 147.4 ± 33.0 and 160.33 ± 28.67 (*P* < 0.001); SF-36 PCS, 45.7 ± 7.8 and 48.7 ± 6.9 (*P* < 0.001); and SF-36 MCS, 45.1 ± 10.5 and 47.2 ± 9.5 (*P* = 0.045). For CD patients, the results were as follows, respectively: IBDQ, 161.1 ± 23.4 and 166 ± 18 (*P* = 0.049); SF-36 PCS, 48.3 ± 6.1 and 50.6 ± 5.3 (*P* = 0.01); and SF-36 MCS: 46.7 ± 8.3 and 48.2 ± 8.1 (*P* = 0.462). QoL changes between pretreatment and posttreatment (12th week) intervals are shown in Table 2 and Figure 2.

There were no serious adverse events causing the cessation of the treatment during or after the IV FeS therapy. Hypotension and flushing occurred in one patient during the infusion, but this was treated with IV antihistamine and saline infusion so that the IV FeS infusion could be completed. There were not any IBD flares or bowel functional disorders related to administration of IV FeS injection.

## 4. Discussion

Iron is an essential element for all living organisms. It is typically paired with hemoglobin and plays pivotal roles in immunological functions, maintaining exercise capacity, and in neurotransmitter metabolism, which maintains cognitive functions such as learning and memory. Furthermore, iron implements these metabolic effects independent of hemoglobin level [2].

There have been many studies investigating the effects of iron replacement therapy in NAID patients [16]. Recently, the positive effects of intravenous iron treatment on QoL in chronic heart failure patients were shown to be independent of anemia [17].

In some studies, the ID ratio in IBD patients has been reported to be as high as 80–90%, which is higher than the anemia ratio in IBD [18]. Unfortunately, usually neither the patient nor the doctor recognizes iron deficiency in IBD since the patient often perceives the symptoms as part of the normal course of the disease [19].

Some studies have shown that the negative impact of anemia on QoL in IBD patients could be reversed by iron treatment. Wells et al. reported a significant improvement in QoL in CD patients with IDA after iron replacement [20]. Evstatiev et al. showed a parallel improvement in QoL and anemia with ferric carboxymaltose (FCM) in the FERGIcor study [21].

In 2007, Gasche et al. published guidelines for ID in IBD, and following this, the European Crohn's and Colitis Organisation (ECCO) announced guidelines for the description and treatment of anemia in IBD [22, 23]. Despite these guidelines, there is still no consensus on the management of NAID, which is a situation (described as normal hemoglobin but with low ferritin or TfS levels) often seen in daily clinical practice, especially in the IBD remission phase. These patients are typically not treated with iron since their hemoglobin levels are normal and there are concerns about oral iron preparations having negative side effects (such as gastrointestinal intolerance or Fenton reaction) and intravenous iron preparations have caused fatal allergic reactions in the past. In the FERGImain study, patients treated for anemia were followed up for 8 months, and those with ferritin levels lower than 100 *μ*g/L were retreated with FCM. Results of that study indicate that iron replacement decreased the recurrence of anemia. The same study also showed that intravenous iron replacement improved QoL, but this improvement was not significant [24].

In our study, intravenous iron treatment in NAID patients with IBD improved iron parameters and QoL. There were statistically significant improvements in both IBDQ and SF-36 for UC and CD patients, although a subgroup analysis indicated that those with UC had a more significant increase in QoL than did patients with CD. In addition, after intravenous FeS treatment, the SF-36 MCS scores of CD patients did not increase significantly. This may be because the CD group had fewer patients or because CD is a more debilitating disease than UC.

Our study has some limitations. There are some differences between our study and the other similar investigations in the literature. Some studies regarding anemia and ID in IBD estimate that iron deficiency during NAID and remission is as high as 600–1000 mg when adjusted for gender and weight [25]. Therefore, we could have given more than 500 mg iron in our study to obtain higher blood iron levels. Additionally after IV FeS administration, QoL could have been assessed earlier than 12th week such as 4th and 8th weeks and the results could have been compared. In addition, new intravenous iron agents that can be administered more easily, including FCM and iron isomaltoside, are now available and should be used in further QoL studies.

In conclusion, ID is a prevalent problem in IBD that can decrease QoL independent of hemoglobin levels. Patients with IBD can become anemic or ID any time throughout the course of the disease. Because of this, IBD patients should be periodically evaluated for anemia and ID since they can commonly reoccur. Iron replacement therapy should be kept in mind in IBD patients with NAID.

## Figures and Tables

**Table 1:** Descriptive statistics of demographic variables (*n* = 85).

Age (mean, ±SD)	42.2	13.1
Sex (*n*, %)		
Male	46	54.1
Female	39	45.9
Disease type (*n*, %)		
UC	55	64.7
CD	30	35.3
Disease duration (median, IQR)	4.0	6.0
UC localization (*n*, %)		
Extensive	25	45.5
Left-sided	22	40.0
Proctitis	8	14.5
CD localization (*n*, %)		
Ileal	11	36.7
Colonic	5	16.7
Ileocolonic	13	46.6
CD type (*n*, %)		
Inflammatory	23	76.7
Stricturing	4	13.3
Penetrating	3	10.0

UC: ulcerative colitis, CD: Crohn's disease, and IQR: interquartile range.

**Table 2:** Comparison of quality of life parameters before and after intravenous FeS treatment.

	Baseline	Follow-up	*P*
	Mean ± SD	Mean ± SD	
Total (*n* = 85)			
IBDQ	152.3 ± 30.6	162.3 ± 25.5	<0.001
SF-36 PCS	46.7 ± 7.3	49.3 ± 6.4	<0.001
SF-36 MCS	45.7 ± 9.8	47.6 ± 8.9	0.024
UC (*n* = 55)			
IBDQ	147.4 ± 33.0	160.33 ± 28.7	<0.001
SF-36 PCS	45.7 ± 7.8	48.7 ± 6.9	<0.001
SF-36 MCS	45.1 ± 10.5	47.2 ± 9.5	0.045
CD (*n* = 30)			
IBDQ	161.1 ± 23.4	166 ± 18	0.049
SF-36 PCS	48.3 ± 6.1	50.6 ± 5.3	0.010
SF-36 MCS	46.7 ± 8.3	48.2 ± 8.1	0.462

FeS: iron sucrose, SD: standard deviation, IBDQ: Inflammatory Bowel Disease Questionnaire, SF: short form, PCS: Physical Component Summary, MCS: Mental Component Summary, UC: ulcerative colitis, and CD: Crohn's disease.

## Abbreviations

IBD: Inflammatory bowel disease
IBDQ: Inflammatory Bowel Disease Questionnaire
ID: Iron deficiency
IDA: Iron deficiency anemia
CD: Crohn's disease
ECCO: European Crohn's and Colitis Organisation
FCM: Ferric carboxymaltose
FeS: Iron sucrose
Hb: Hemoglobin
NAID: Nonanemic iron deficiency
MCS: Mental Component Summary
PCS: Physical Component Summary
QoL: Quality of life
SF-36: Short form-36
SPSS: Statistical package for the social sciences
TfS: Transferrin saturation
UC: Ulcerative colitis.
